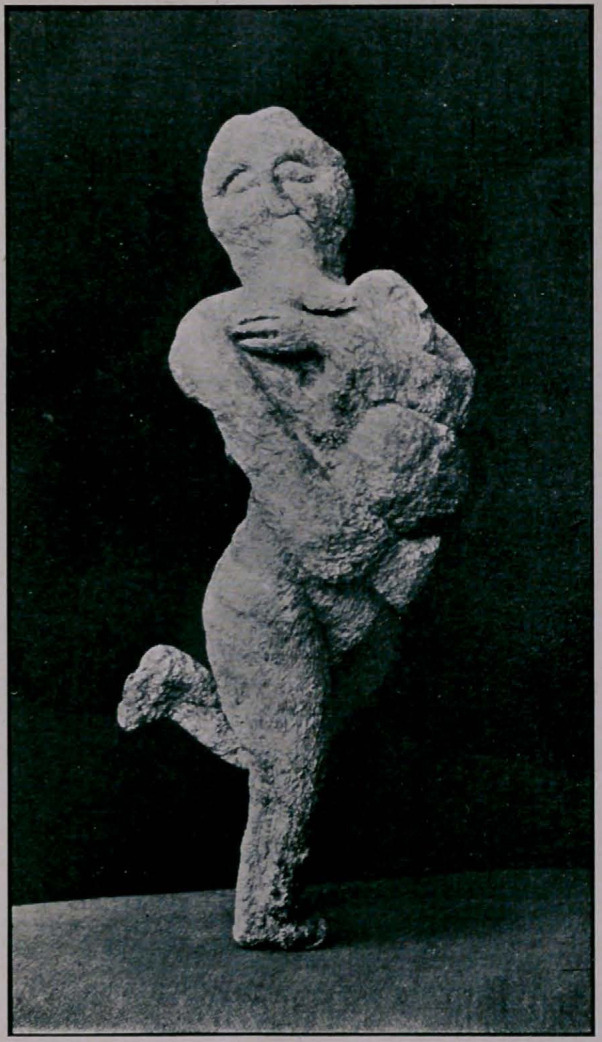# The Mandrake of the Bible

**Published:** 1903-04

**Authors:** 


**The Mandrake of the Bible.**

(*Mandragora Officinarum.*)

SPECIMEN FOUND IN SILICIA.

"And Reuben went in the days of wheat harvest, and found mandrakes in the field, and brought them unto his mother, Leah. Then Rachel said to Leah, 'Give me, I pray thee, of thy son's mandrakes.'

"And she said unto her, 'Is it a small matter that thou hast taken my husband? And wouldst thou take away also my son's mandrakes?' And Rachel said, 'Therefore he shall lie with thee tonight for thy son's mandrakes.'

"And Jacob came out of the field in the evening, and Leah went out to meet him, and said, 'Thou must come in unto me, for surely I have hired thee with my son's mandrakes.' And he lay with her that night."—Genesis xxx, 14, 15, 16.

(See Daniel's "Recollections of a Rebel Surgeon.")

SOME MORE ABOUT MANDRAKES.

MIDLAND, TEXAS, March 5, 1903.

*Dr. F. E. Daniel, Austin, Texas.*

DEAR DOCTOR: A long time ago I read a very interesting article in the *Texas Medical Journal* entitled, "Dr. Merriman on Mandrakes." Some time ago my sister, living in Lexington, Ky., knowing my fondness for anything odd, especially if it pertains to the medical world, sent me a photograph of a mandrake root. I wrote back and had her send me another one in order that I might forward it to you. I remember that your "Old Doctor" told you as he left to let him know if you found out anything more "about those mandrakes," hence I suppose you would be further interested, and today I mail you, under separate cover, a photograph that is taken from a genuine specimen. The enclosed letter, by the eminent Prof. McGarvey of the Kentucky Bible College, explains itself. With best wishes, I am yours fraternally,

W. K. CURTIS, M. D.

## Figures and Tables

**Figure f1:**